# Resurgence of Sexually Transmitted Hepatitis C Virus Infection in Men Who Have Sex With Men Living With HIV

**DOI:** 10.1002/jmv.71185

**Published:** 2026-10-17

**Authors:** Shui‐Shan Lee, Denise Pui‐Chung Chan, Bonnie Chun‐Kwan Wong, Danny King Fai Cheung, Ngai Sze Wong

**Affiliations:** ^1^ S.H. Ho Research Centre for Infectious Diseases The Chinese University of Hong Kong Hong Kong China; ^2^ Centre for Health Protection Hong Kong Government Department of Health Hong Kong China; ^3^ JC School of Public Health and Primary Care The Chinese University of Hong Kong Hong Kong China

**Keywords:** HCV, hepatitis C, MSM, sexual transmission

## Abstract

In 2023–2025, HCV transmission in men who have sex with men (MSM) living with HIV in Hong Kong resurged. This study aims to assess the epidemiologic patterns between 2011 and 2025. Among 288 MSM with sexually acquired HCV infection, 73% were diagnosed at least 12 months after HIV diagnosis, and 98% had ever achieved HIV viral load suppression. Comparing with Period 1 (2015–2022) featuring a large HCV genotype 3a cluster (89 diagnoses) and a few smaller HCV genotype 1a clusters (size of 2 to 13), Period 2 (2023–2025) was dominated by two HCV genotype 1a clusters (7 and 16 new diagnoses). Transmission of genotype 3a viruses subsided in Period 2, with only 3 new diagnoses. Comparing with Period 1, a higher proportion of MSM with HCV diagnoses in Period 2 self‐reported sexualised injecting drug use (OR = 3.82, 95% CI 1.59–9.20) and HCV direct‐acting antivirals therapy (no treatment as reference group, OR = 3.72, 95%CI 1.82–7.60). The epidemic rebound by a different HCV genotype was likely contributed by increased engagement in chemsex and post‐COVID‐19 population mobility.

## 1 Introduction

Hepatitis C virus (HCV) is a member of family *Flaviviridae*. Classified into eight genotypes and with over 90 subtypes, the diversity of HCV has expanded over years and geographically [1]. Previously considered a predominantly blood‐borne infection, sexual transmission of HCV has emerged among men who have sex with men (MSM) since the turn of the millennium, as reported in western Europe, the United States, and the Asia Pacific [2]. People living with HIV constitute a unique community group with high infection burden [3].

With the advent of direct‐acting antivirals (DAA) since the 2010s, the epidemics of sexually transmitted HCV in MSM living with HIV have shown signs of abatement. Declines of HCV viraemia have been reported in high‐income countries [3], notably US [4], United Kingdom [5], Germany [6] and also Taiwan in the Asia Pacific [7]. This study reports the resurgence of the epidemic in Hong Kong, following similar declines worldwide in the early 2020s. In this study, we assessed the epidemiologic patterns of sexually acquired HCV infection in MSM living with HIV.

## 2 Materials and Methods

Clinical data of patients attending the Integrated Treatment Centre who had been diagnosed with acute HCV infection were retrospectively collected. The Centre is the largest clinical HIV treatment service in the city of Hong Kong, serving over 4000 patients living with HIV [8]. Regular screening and symptom‐based opportunistic testing for HCV are provided to MSM living with HIV at the Centre, following which contemporary standard of care HCV therapy (including IFN‐based treatment or DAA) was prescribed for people diagnosed with viraemic infection. In Hong Kong, DAA accessibility was unrestricted, and has been enhanced through expanding the treatment indication from advanced fibrosis to F2 in 2019, and to treat all in 2021.

The clinical data included socio‐demographics (year of birth, gender, local residency), HIV status (date of HIV diagnosis, history of AIDS diagnosis, mode of HIV transmission, year of starting HIV antiretroviral therapy (ART), ART regimen at HCV diagnosis, nadir CD4 count, HIV viral load suppression), HCV condition (date of HCV diagnosis, history of previous HCV diagnosis, HCV genotype, HCV treatment regimen and date, HCV molecular clusters), measurements (HIV RNA, CD4 count, HCV RNA, highest alanine aminotransferase (ALT) at HCV diagnosis), concurrent sexually transmitted infections (STIs), and sexual behaviour (chemsex engagement as defined as sexualised use of methamphetamine and/or GHB [8]; and sexualised injecting drug use). HCV molecular clusters (> 2 connected nodes) and pairs (2 connected nodes) were identified by phylogenetic analysis of typable NS5B region of HCV genome. Bootstrap value above 70% (1000 replicates) was considered as significant.

Based on the annual number of acute HCV diagnoses, two periods were delineated. To compare the characteristics of the two periods, if an individual had more than one HCV diagnoses in the period, the earlier HCV diagnosis record was selected for analysis. The two periods were compared in bivariable logistic regression model in IBM SPSS Statistics 30. Complete‐case analyses were performed. The study was approved by Joint Chinese University of Hong Kong – New Territories East Cluster (CUHK‐NTEC) Clinical Research Ethics Committee (approval number: CREC2015.320). Informed consent was waived. Results of the study on the epidemiology of the HCV co‐infection as of 2021 had been published [9].

## 3 Results

### 3.1 Characteristics of the Study Population

Between 2011 and 2025, a total of 327 episodes of acute HCV infections were diagnosed among MSM living with HIV. Figure 1 shows the gradual increase in the yearly number of diagnosed HCV infections rising from at most 10 in and before 2013 to a peak of 34 in 2018. It then plateaued off and there was a steep decline to just 8 cases in 2023. In the subsequent years, the epidemic resurged with a peak of 37 cases in 2025. High number of acute HCV infection continued to be noted in the first half of 2026 (data not shown).

**Figure 1:**
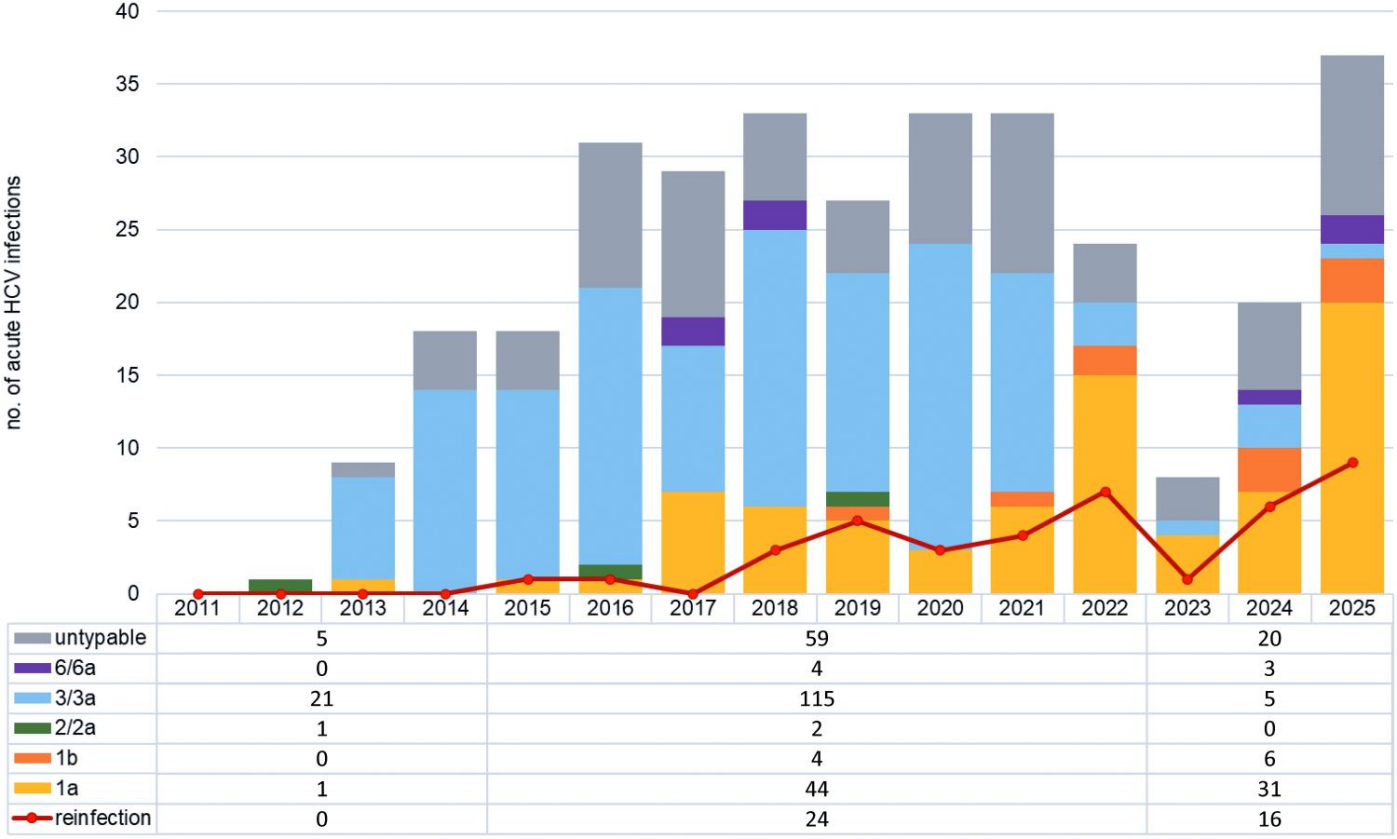
Yearly number of acute hepatitis C (HCV) infection in men who have sex with men (MSM) living with HIV, 2011–2025.

Among 288 MSM living with HIV diagnosed with acute HCV infection in 2011–2025, the median age at HCV diagnosis was 36 years (IQR: 30 to 42) and 99% were local residents (Table 1). The median number of months from HIV to HCV diagnosis was 44 (IQR: 10 to 94), with three‐quarters (73%) having had HIV diagnoses made over 12 months prior to HCV diagnoses. Almost all (98%) had ever achieved HIV viral load suppression. The median nadir CD4 count was 288 cells/µL (IQR: 172–400), while that at HCV diagnosis was 545 cells/µL (IQR: 380–714). A majority (88%) had never been diagnosed with AIDS. All except 5 (98%) were on antiretroviral therapy at the time of HCV diagnosis, of which 64% were on integrase strand transfer inhibitor (INSTI)‐based regimen.

**Table 1:** General characteristics of MSM living with HIV who were diagnosed with acute HCV infection in 2011–2025 (*N* = 288).

	*n*/median	%/(IQR)
Baseline characteristics		
Local residency^a^		
No	2	1%
Yes	282	99%
Age at HCV diagnosis (years)	36	(30–42)
Months from HIV diagnosis	44	(10–94)
Clinical HIV status		
Never diagnosed with AIDS defining illness	253	88%
Ever achieved viral load suppression	281	98%
Nadir CD4 (cells/μL)	288	(172–400)
< = 350	185	64%
351–500	60	21%
> 500	43	15%
On antiretroviral therapy^b^	281	98%
CD4 count (cells/µL) at first HCV diagnosis^c^	545	(380–714)
< = 350	53	19%
351–500	70	25%
> 500	156	56%
Condition at first HCV diagnosis in the period		
Previous HCV diagnosis		
No	287	99.7%
Yes	1	0.3%
Year of HCV diagnosis	2019	(2016–2021)
HCV RNA (log_10_ copies/mL)^d^	6.39	(5.32–6.88)
Highest ALT (IU/L)^e^	216	(83–506)

Abbreviation: ALT, Alanine Aminotransferase.

^a^ 4 missing.

^b^ 2 missing.

^c^ 9 missing.

^d^ 63 missing.

^e^ 8 missing.

### 3.2 Comparison Between Two Periods

Two time periods could be delineated: Period 1 from 2015 to 2022 (*n* = 209); Period 2 from 2023 to 2025 (*n* = 59), which is still ongoing. Patients in Period 2 had a longer median interval from HIV diagnosis (100 vs 39 months, OR 1.01) and higher median CD4 count (650 vs 545 cells/µL, OR 1.002) at HCV diagnosis (Table 2). A significantly higher proportion of patients in Period 2 had presented with reinfection (first reinfection 12% vs 1%, OR 14.79, 95%CI 2.98–73.38) and DAA therapy (81% vs 48%, OR 3.72, 95%CI 1.82–7.60, no treatment as reference group). There were 237 typable HCV sequences (the remaining were untypable due to low viral load) from the cohort: 1a (76, 32%), 1b (10, 4%), 2/2a (3, 1%), 3/3a (141, 59%), 6/6a (7, 3%) (Figure 1). The distribution of the HCV genotypes varied between the 2 periods. Among typable samples, whereas genotype 3/3a had dominated in Period 1, genotype 1a was dominant in Period 2.

**Table 2:** Comparison of characteristics of diagnosed acute HCV infection between Period 1 (2015–2022) and Period 2 (2023–2025).

	Total (*N* = 268)	2015–2022 (*N* = 209)		2023–2025 (*N* = 59)		Logistic regression	
	*n*	*n*	%	*n*	%	OR	95%CI
HCV diagnosis and clinical course							
Median age (years) (IQR)	35 (30–42)	35	(29–42)	39	(33–47)	/	
Median year of HCV diagnosis (IQR)	2020 (2017–2022)	2018	(2017–2020)	2025	(2024–2025)	/	
HCV reinfection							
No	256	207	99%	49	83%	Ref	
First reinfection	9	2	1%	7	12%	14.79*	(2.98–73.38)
Second reinfection	3	0	0%	3	5%	/	
Median HCV RNA (log_10_ copies/mL) (IQR)^a^	6.39 (5.33–6.88)	6.51	(5.43–6.94)	5.86	(4.75–6.61)	0.80	(0.63–1.01)
Median highest ALT (IU/L) (IQR)^b^	193.5 (77.25–481.5)	195	(81–463)	192	(55–572)	1.000	(0.999–1.001)
HCV treatment							
No	97	86	41%	11	19%	Ref	
DAA	149	101	48%	48	81%	3.72*	(1.82–7.60)
IFN based	22	22	11%	0	0%	/	
HIV status at HCV diagnosis							
Median months from HIV diagnosis (IQR)	49 (9–103)	39	(7–87)	100	(51–127)	1.01*	(1.004–1.01)
> 12 months	196	148	71%	48	81%	Ref	
≤ 12 months	72	61	29%	11	19%	0.56	(0.27–1.14)
Median months from ART start (IQR)^c^	13 (1–42)	10	(1–32)	43	(6–59)	1.02*	(1.01–1.03)
ART regimen							
INSTI	183	135	65%	48	81%	7.82*	(1.83–33.51)
NNRTI	34	27	1%	7	3%	5.70*	(1.10–29.49)
PI	46	44	13%	2	12%	Ref	
Not on ART	5	3	21%	2	3%	14.67*	(1.50–143.73)
Median CD4 count (cells/μL) (IQR)^d^	559 (392–726)	545	(372–692)	650	(475–811)	1.002*	(1.001–1.003)
< = 350	46	38	19%	8	15%	Ref	
351–500	61	51	25%	10	19%	0.93	(0.34–2.58)
> 500	151	115	56%	36	67%	1.49	(0.64–3.48)
Sexual behaviour at HCV diagnosis							
Chemsex engagement^e^							
No	221	175	84%	46	79%	Ref	
Yes	45	33	16%	12	21%	1.38	(0.66–2.89)
Sexualised injecting drug use^e^							
No	243	196	94%	47	81%	Ref	
Yes	23	12	6%	11	19%	3.82*	(1.59–9.20)
Concurrent STI							
No	88	50	24%	38	64%	Ref	
Yes	180	159	76%	21	36%	0.17*	(0.09–0.32)

*Note:* Chemsex engagement – any use of methamphetamine, GHB/GBL, and mephedrone during sex during the time period that HCV transmission presumable occurred; Concurrent STI – diagnosis of STI within one year of HCV diagnosis.

Abbreviations: ALT – Alanine Aminotransferase; ART – HIV anti‐retroviral therapy; CI – confidence interval; IQR – interquartile range; OR – odds ratio; STI – sexually transmitted infection

^a^ 60 missing.

^b^ 8 missing.

^c^ 3 missing.

^d^ 10 missing.

^e^ 2 missing.

* *p* < 0.05.

Molecular clustering of cases was observed in both periods but in distinctly different patterns (Figure 2). A genotype 3a cluster with 89 diagnoses in 2013–2022 [10] has subsided to just 3 new diagnoses in 2023–2025 (Period 2). While genotype 1a pair/clusters in Period 1 were smaller (size of 2 to 13), two clusters (Cluster2 and Cluster5) grew rapidly in Period 2. The size of Cluster2 grew from 13 in Period 1 to 29 in Period 2, including 16 new HCV diagnoses. Cluster5 increased from 5 to 12 in the period (7 new diagnoses). Sexualised injecting drug use was more commonly reported by cases in Period 2 compared to Period 1 (OR 3.82, 95% CI 1.59–9.20) (Table 2). Concurrent STI was however less prevalent in Period 2 compared to Period 1 (36% vs 76%, OR 0.17, 95% CI 0.09–0.33).

**Figure 2:**
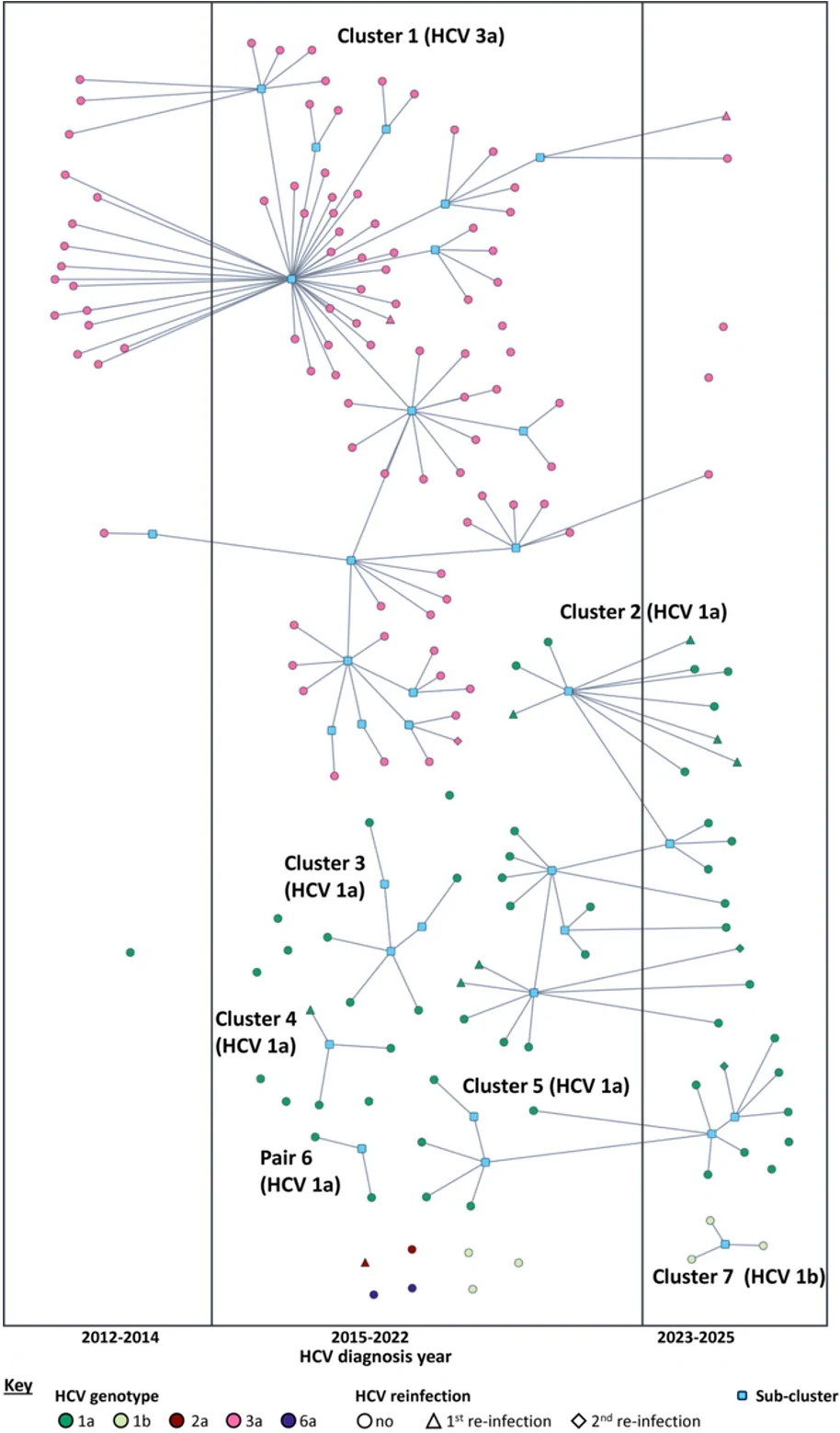
Clustered transmission of acute HCV infections in MSM living with HIV in Hong Kong between 2012 and 2025 (*N* = 167). as illustrated by the distribution of 6 monophyletic clusters (> 2 samples) and 1 pair (bootstrap value > 70%) from phylogenetic analysis of typable NS5B sequences from the study cohort – Cluster 1 (genotype 3a); Cluster 2 to Cluster 5 (genotype 1a); Pair 6 (genotype 1a); Cluster 7 (genotype 1b). The clustering pattern is illustrated in 2‐mode network (infection episode and subcluster).

## 4 Discussion

Our analyses illustrated the HCV transmission dynamics in MSM living with HIV in Hong Kong, the epidemiologic pattern of which was very similar to that in other parts of the world, as characterised by a peak in the 2010s followed by a significant decline in early 2020s [4, 5, 6, 7]. Increased testing and wide access to effective DAA treatment had contributed to the steep decline [11]. In our study, HCV genotype 3a dominating Period 1 had almost disappeared by 2023. The drop of HCV cases in 2021–2023 could be partly due to COVID‐19 lockdown causing temporary closure or operation restrictions to venues such as gay bars and saunas in 2020–2022 [12] and changes in sexual behaviours, in line with the global decline of STI reports [13]. The worldwide declines of sexually transmitted HCV in MSM living with HIV appeared to have brought us close to microelimination [3], but only temporarily. Given the absence of protective immunity and the lack of means of prophylaxis, MSM living with HIV are liable to be exposed or re‐exposed to HCV through their practice of higher risk sexual behaviours [14].

While the pre‐COVID‐19 HCV epidemics have been widely reported in countries around the world, post‐COVID‐19 resurgence appear to be less common in the literature. Despite the effectiveness of DAA in curing HCV infection, transmission would likely continue unless the virus is eliminated from the community. A systematic review concluded with the occurrence of HCV re‐infection at an incidence of 3.76 per 100 person‐years of follouwp in people living with HIV [15]. A Spanish cohort study reported that re‐infection had accounted for 17.4% of MSM diagnosed with acute HCV infection between 2023 and 2025 [16]. Our results in Hong Kong, a metropolitan Asia‐Pacific city, showed similar resurgence including reinfections in 17%. Routine annual screening has enabled asymptomatic infections to be detected and treatment has been offered without restriction at the same clinic. The subsidence of the genotype 3a dominating outbreaks has however given way to the emergence of genotype 1a outbreaks. As the HIV clinic has continued its service during COVID‐19 with regular HCV screening, the available data did support a true increase in HCV incidence rather than delayed diagnoses after 2023. Clustered transmission could be shown in both periods, though they were caused by a different virus genotype. Clustering reflected MSM's exposure to HCV in venues or activities participated by people in high burden communities. Indeed the Period 2 in our study was associated with chemsex and especially sexualised injecting drug use, both of which similarly reported in Spain [15]. The lifting of COVID‐19 lockdown had led to rebounds as a result of increased travels between and within cities/countries. The linkage of HCV between cities/countries could be uncovered by molecular studies on sequences from different places [10].

There are several limitations in the study. First, data were collected from one of three HIV specialist clinics in Hong Kong, the results of which might not be fully representative as other transmission chains may present. Second, we acknowledge the potential HCV linkage with their partners who were not MSM, although previous study has suggested distinctive epidemiologic differences between MSM and non‐MSM on HIV/HCV co‐infections [9]. Similarly, the HCV linkage of MSM living with HIV and MSM without HIV were not included, although local exploration only identified very limited number of sexually transmitted HCV infection in people without HIV. Third, the description of genotype clusters over time was limited to typable samples, and results should be interpreted with caution.

## 5 Conclusions

In conclusion, sexually transmitted HCV genotype 3a dominating outbreaks in MSM living with HIV in Hong Kong had declined during COVID‐19, which was replaced by an epidemic rebound by a different HCV genotype, precipitated by increased mobility of people after lifting of lockdown and increased engagement in chemsex.

## Author Contributions

S.S.L. and N.S.W. contributed to the conception and design of the study. D.P.C.C. and N.S.W. did the data analyses. B.C.K.W. and D.K.F.C. collected the clinical data and contributed to data interpretation. S.S.L. wrote the first draft of the manuscript, all contributed to its revision and final approval of the manuscript.

## Ethics Statement

The study was approved by Joint Chinese University of Hong Kong – New Territories East Cluster (CUHK‐NTEC) Clinical Research Ethics Committee (approval number: CREC2015.320). Informed consent was waived.

## Conflicts of Interest

The authors declare no conflicts of interest.

## Data Availability

The individual‐level dataset analysed in this study cannot be shared publicly because the data are owned by third parties. Access to these data and permission could be inquired through the Department of Health, Hong Kong SAR Government.
